# Development of a nurse-manager dualistic intervention program to alleviate burnout among nurses based on the appreciative inquiry

**DOI:** 10.3389/fpsyg.2022.1056738

**Published:** 2022-12-06

**Authors:** Yu-Fang Guo, Xin-Xin Wang, Fang-Yan Yue, Feng-Ye Sun, Min Ding, Yan-Nan Jia

**Affiliations:** ^1^School of Nursing and Rehabilitation, Shandong University, Jinan, Shandong, China; ^2^Clinical Psychology Department, Peking University Sixth Hospital, Beijing, China; ^3^Department of Critical Care Medicine, Shandong Provincial Hospital Affiliated to Shandong First Medical University, Jinan, Shandong, China; ^4^Committee of the Communist Youth League, Shandong University, Jinan, Shandong, China

**Keywords:** appreciative inquiry, burnout, formative intervention development, nurse, qualitative research, quantitative research

## Abstract

**Aims:**

To develop a feasible and effective nurse-manager dualistic intervention program to support nurses coping with burnout symptoms.

**Background:**

Person-organization combined interventions were recommended as the most effective approach for reducing burnout symptoms. However, few interventions have been developed in the nursing field.

**Methods:**

The Medical Research Council, United Kingdom (MRC UK), Framework for Development and Evaluation of Complex Interventions, was employed for nurse-manager dualistic intervention program development. The following three steps were followed for developing the dualistic intervention program: (1) identifying the evidence base by conducting extensive reviews of the relevant literature and a mixed study; (2) identifying/developing a theory by selecting the job demands-resources model and proposing the theoretical framework for intervention development; and (3) modifying the process and outcomes of the nurse-manager dualistic intervention program.

**Results:**

The intervention program consists of six group sessions over 9 weeks. Researchers/managers are supposed to deliver the program. The main contents of the intervention are (1) inception (session 1); (2) discovery (session 2); (3) dream (session 3); (4) design (session 4); (5) destiny (session 5); and (6) keep (session 6). The emphasis of the intervention is on helping nurses dealing with burnout symptoms.

**Conclusion:**

Following the guidance of the MRC framework, a feasible and potentially effective nurse-manager dualistic intervention program was developed for nurses coping with burnout. Future studies are needed to model the intervention and assess the effects and replicability of the intervention.

## Introduction

Burnout is a psychological response to chronic work-related stress that is characterized by emotional exhaustion, cynicism, and reduced professional accomplishment. According to Leiter and Maslach (2004), a chronic mismatch between a person and an organization plays a key role in burnout development. The severe conflict between personal and organizational values negatively influences job involvement, decision-making participation, and organizational identification, which, in turn, leads to burnout (Leiter and Maslach, 2004). As one of the most important psychological occupational hazards, burnout develops progressively and negatively affects work attitude and behavior, which causes damages at a personal (e.g., poor work performance, turnover intention, and absence) and organizational level (e.g., low organizational commitment, poor peer supports, and substantial financial costs) (Fish et al., 2022; Li et al., 2022; Shapiro et al., 2022). A growing body of empirical evidence shows that burnout prevalence is high in healthcare professions, especially in the nursing group, with 20%−80% of nurses acknowledging severe burnout symptoms worldwide (Matsuishi et al., 2021; Membrive-Jimenez et al., 2022; Wei et al., 2022). Therefore, it is urgent to develop effective intervention strategies to alleviate nurse burnout, which ultimately contributes to high levels of work passion, work performance, psychological wellbeing, and organizational commitment.

## Background

Since the 1980s, numerous studies have been conducted to figure out effective intervention approaches that prevent and alleviate burnout symptoms. According to types, purposes, and target populations of the interventions, researchers normally classify the burnout interventions into one of the following three broad categories: (1) person-directed interventions aim to reduce individuals' burnout experience, usually through promoting their coping skills, acquiring supports from family and organization, or changing negative cognition and behaviors (Dreison et al., 2018); (2) organization-directed interventions concentrate on modifying the organization and job characteristics related to burnout, such as negative work environment, inadequate organizational resources, injustice, and work overload (Edu-Valsania et al., 2022); (3) person-organization combined interventions focus on promoting coping strategies of individuals and employee-driven organizational change *via* interaction of individuals with organizations and aspects of their job (Awa et al., 2010).

Typical person-directed interventions mainly include classic cognitive-behavioral therapies (e.g., emotional self-regulation, cognitive restructuring, and relaxation), mindfulness training, and physical exercise. Most of the interventions, presented in a workshop or online software, were initiated and determined by the employees themselves, to strengthen their psychological and behavioral states, which were completely outside the organizational problems (Schaufeli and Enzmann, 1998). For example, Bagheri et al. (2019) employed a stress-coping strategy and group cognitive-behavioral therapy on clinical nurses and found that the intervention significantly reduced nurse burnout and the effectiveness lasted after a month. Duarte and Pinto-Gouveia (2016) provided a mindfulness-based intervention on oncology nurses and found a significant decrease in burnout, compassion fatigue, and stress and a significant increase in life satisfaction and self-compassion, with medium to large effect sizes. Semerci et al. (2021) used progressive muscle relaxation exercises on burnout and compassion of nurse managers and found that the intervention significantly alleviates burnout and compassion fatigue of managers.

A further narrative review identified several studies that showed no effect of person-directed interventions on nurse burnout. The nurse-led cognitive-behavioral therapy analyses by Partlak Gunusen et al. (2021) and mindfulness analyses by Wong et al. (2021) did not show any positive effects on nurse burnout. Taken together, since burnout develops idiosyncratically due to person and organization mismatch, the negative effects of organizational conditions on burnout have not been involved in person-directed interventions. Moreover, the effectiveness of person-directed interventions on nurse burnout reduction needs further evidence to be proven. In addition, concerns about the limited long-term effects of person-directed interventions have been raised by researchers and managers (Jensen et al., 2006; Westermann et al., 2014).

Organization-directed interventions involve developing welcoming programs, using rewards and incentives, implementing work-family balance plans and humanizing work schedules, improving job characteristics and leadership of managers, training and coaching employees, and creating supportive environments (Shanafelt and Noseworthy, 2017; DeChant et al., 2019). Kersten et al. (2019) used a dialysis-specific training program (including healthy behavior management, promotion of work characteristics, and organizational resource identification and enhancement) to reduce nurse burnout and found that burnout had improved after the intervention, although the effects were not stable over time and the effect sizes were small. Wei et al. (2017) developed an active intervention, including communication skills, conflict coping strategies, efficacy elevation, working skills, and emotion management, and conducted a randomized control trial on burnout nurses. They found significant decreases in emotional exhaustion and depersonalization in nurses. Kelly et al. (2012) explored the effect of creating a positive organizational culture *via* Magnet hospital on burnout and found that nurses from Magnet hospital reported lower burnout symptoms than nurses from the non-Magnet hospital. Despite several organization-directed intervention studies reporting positive findings, these interventions do not target specific personal issues, which might lack feasibility for individuals and hardly motivate their subjectivity (Tsutsumi et al., 2009). Furthermore, difficulties in repetitive verification and challenges in the popularization and application of the interventions make it hard to conclude (Dreison et al., 2018).

Person-organization combined interventions are multifaceted, initiated, and determined by both employees and managers. The typical interventions often use stress management with organizational environment improvement (e.g., stress coping strategies with organizational support and psychotherapy with job crafting and time management). A previous systematic review showed that combined intervention approaches were completely different in terms of content, form, duration, and frequency (Pijpker et al., 2019). Adams et al. (2019) used a 2-month cultural change toolkit, including meaningful recognition, shared decision-making, and increased leadership involvement and support, to promote nurse burnout and found a significant burnout reduction after the implementation. Liu et al. (2022) provided a 1-year rational emotional intervention combined with hierarchical management to burnout nurses and found that nurses experienced lower levels of burnout after the intervention. Given the comprehensiveness of combined interventions, it is recommended as the most effective intervention type. However, most of the combined interventions focus on stress reduction and work condition promotion, which hardly tackles the burnout root of the person and organization mismatch issue. In addition, few interventions simultaneously motivate personal strengths and organizational resources to cope with burnout adversity, which impacts the positive attributes and organizational involvement of nurses.

Appreciative inquiry (AI), developed by Cooperrider (1986), is based on a relational constructionist view that places a strong emphasis on human perceptions, social collaboration, and appreciative systems. As a new organizational development intervention, AI seeks innovative ideas from employees and searches for success for employees and their organizations *via* affirmation, appreciation, and dialogue (Koster and Lemelin, 2009). AI advocates a positive slant of inquiry based on future possibilities instead of problem-based short-term solutions and focuses on positive changes arising from the interaction between employees' language, relationships, and functioning in an organization (Cooperrider and Whitney, 2000, 2005). There are four phases of inquiry cycles that form the process of AI: (1) discovery uses emotional touchpoints and photoelicitation to discover what is working well and appreciate what is the best of what has been; (2) dream envisions what would be the ideal dream for the future; (3) design co-constructs a vision for the ideal future; and (4) destiny plans the work strategies toward the desired vision. Through this 4D (discovery, dream, design, and destiny) cyclical process, AI provides a flexible framework for discovering and utilizing personal strengths and organizational resources to achieve organizational goals. AI has been widely employed in several fields, such as education (Stulz et al., 2021), organization management (Martyn et al., 2019), and healthcare promotion (Shrivastava et al., 2020), and is proven to be an effective method in generating personal growth (Sturm et al., 2020), leadership promotion (Bleich and Hessler, 2016), environment change (Ebert et al., 2020), capacity building (Magnussen et al., 2019), and organizational development (Hilde et al., 2010).

Therefore, taking the intrinsic causes of burnout and the benefits of person-organization combined interventions into account, this study aimed to employ the 4D cycle of AI to develop an AI-based nurse-manager dualistic intervention program to support burnout nurses, guided by the MRC framework for developing and evaluating complex interventions.

## Methods

This was an interventional development study. The framework for the development and evaluation of complex interventions developed by MRC, UK, was employed as a stepwise guideline for developing the nurse-manager dualistic intervention program. There are four stages of the MRC framework, namely, development, feasibility/piloting, evaluation, and implementation. In this study, we just reported the development stage of the AI-based nurse-manager dualistic intervention (NMDI) program. The following three steps of the development stage were followed: identifying the evidence base, identifying/developing theory, and modifying processes and outcomes. Table 1 shows the activities we undertook for developing an AI-based NMDI program following the guidelines of the MRC. This study was approved by the Human Research Ethics Committee of the School of Nursing and Rehabilitation, Shandong University (NO. 2020-R-030). Approvals were gained from the two tertiary hospitals in Baoding where the study was carried out.

**Table 1:** Activities for developing NMDI program following the guidelines of the MRC.

**Steps for developing a complex intervention following the MRC framework**	**Activities taken to develop NMDI program**
Identifying the evidence base	(i) Identifying existing reviews of burnout interventions (ii) Conducting extensive reviews of studies related to appreciative inquiry interventions in nursing group (iii) Primary research: a mixed study were conducted to evaluate the attitudes and suggestions of nurses and nurse managers toward burnout intervention
Identifying/developing theory	(i) Literature review and team discussions to choose an appropriate theory and intervention strategies
Modifying process and outcomes	(i) Developing the contents of the NMDI program. As to optimize the intervention, an iterative process was employed with involvement of nursing experts, management experts, nurse managers and nurses

## Results

### The identified evidence base

According to the framework of the MRC, we first identified the evidence base *via* a systematic review and a mixed study.

#### Review of interventions to alleviate burnout

A systematic review and meta-analysis of burnout interventions identified 29 studies (Dreison et al., 2018). Of these studies, 21 were organizational-directed interventions, with the subtypes of job training and education, coworker support groups, and clinical supervision; six were personal-directed interventions, and the most common subtype was a stress management workshop; and only two were personal-organizational combined interventions, with the subtypes of a stress management workshop, ongoing workgroups, and organizational consultation. Studies indicated that combined interventions, targeting both personal and organizational factors, were the most effective strategies for decreasing burnout symptoms (Awa et al., 2010; Morse et al., 2012).

#### Review of appreciative inquiry interventions in the nursing group

To increase our understanding of AI interventions, we conducted extensive reviews related to AI interventions aimed to improve the emotions, behavior, and performance of nurses. These reviews focused on the contents, implementation methods, participants, duration, and outcome measures of interventions. The findings of the review showed that most AI interventions followed the four core components (discover, dream, design, and destiny) to develop the interventions; a group-based method was commonly used to implement the interventions, and nurses were the main participants, with several studies involving managers, doctors, and other health-related professions. The duration of the interventions varied, ranging from 2 days to 2 years. Semi-structured interviews, self-rating questionnaires, work summaries, and work diaries were used as measures to evaluate the work attitude, emotion, behavior, and performance of nurses.

These literature reviews provided some important suggestions on the development of interventions targeting burnout reduction and AI application in nurses: person-organization combined interventions should be developed for alleviating nurse burnout; nurses and nurse managers should be involved in the intervention as a group; four core components of AI should be followed for developing the content of NMDI program; a comprehensive theoretical framework will be needed to guide the development of this multifaceted intervention and evaluate possible change processes.

#### A mixed study evaluating attitudes and suggestions from nurses and nurse managers toward burnout intervention

A semi-structured face-to-face interview was conducted among nurses and nurse managers to obtain a better understanding of nurses and managers coping with burnout and to evaluate their experiences and suggestions. After a conventional content analysis, three themes and six subthemes were identified. The three themes were as follows: needing help to cope with burnout, burnout intervention measures, and intervention environment.

A quantitative study was conducted to investigate the preference of nurses for burnout intervention. A total of 274 nurses were recruited from two tertiary hospitals. The results showed that over half of the nurses selected a group-based (53.6%) or online-to-offline model of intervention (67.9%), and the roles of managers were advisors and supervisors in the intervention (74.5%). Notably, 38.3% of nurses liked to take burnout interventions in hospitals, 46.0% preferred one time per week of the intervention, and 56.6% of nurses suggested that the length of each section for the intervention should be from 1 to 3 h.

Based on the findings, we drew up a primary conclusion on nurses' experiences of coping and living with burnout and made suggestions for developing effective intervention programs to support burnout nurses. This mixed study showed that nurses struggled with chronic burnout symptoms, that few resources can be used to deal with burnout, and that they had strong preferences in burnout intervention models and implementing forms. These findings not only emphasized the need for burnout interventions in a collaborative nurse-manager combined style but also provided us with valuable information that should be considered in the development of burnout interventions.

### Identifying/developing theory

Numerous personal factors (e.g., self-efficacy and self-esteem) and organizational factors (e.g., peer support and job control) are involved in coping with stress and changing existing psychological burnout. Appropriate theories provide an overarching framework for personal and organizational factors that explain why burnout should be targeted by the intervention. Therefore, after a systematic review and a series of team discussions, we chose the job demand-resources model (JD-R) as the theoretical basis for developing our conceptual framework (Demerouti and Bakker, 2011). The model has been shown to predict work-related psychological and behavioral issues in employees (Garcia-Sierra et al., 2016; Hussein, 2018), and it has been used to develop interventions to alleviate stress and promote work engagement (Richard et al., 2012; Makowska-Tiomak et al., 2022).

Bakker and Demerouti (2007) classified two general categories of stress-related factors (job demands and job resources) in JD-R. Job demands are considered the physical, psychological, organizational, and social aspects of the job that require physical and psychological knowledge, skills, and efforts of employees and, therefore, lead to certain physical and psychological costs. Several kinds of job demands have been listed, such as work overload, irregular work arrangements, and an unfavorable working environment. Job resources refer to the physical, psychological, organizational, and social aspects of the job that could be contributing factors for work achievement, job demand reduction, and personal growth. The job resources may be from organizations (e.g., career opportunities, professional training, and salary), the interpersonal relationship (e.g., supervisor, support, and trust), the job position (e.g., role clarity, responsibility, and right), and the task (e.g., task identity, significance, and autonomy). Xanthopoulou et al. (2007) extended the JD-R model by including personal resources (e.g., self-efficacy, organizational-based self-esteem, and optimism) as predictors for exhaustion and work performance. The job demands and resources negatively interact with each other and then affect the development of job-related health impairments and motivation.

**Figure 1:**
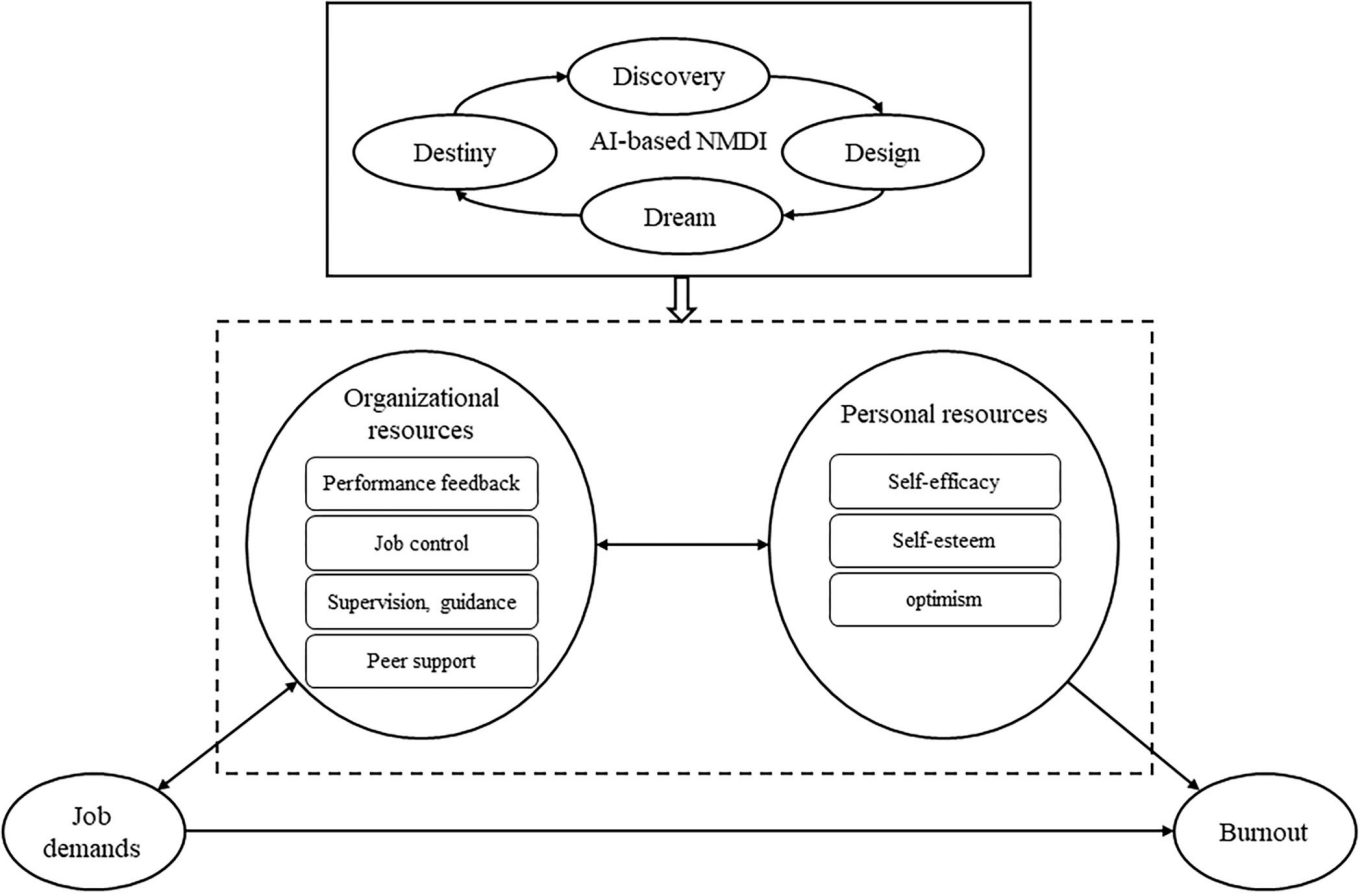
Theoritical framework for AI-based NMDI program targeting in burnout reduction.

In the development of motivation and reduced health/energy, two different underlying psychological processes were found. First, long-term, chronic job demands lead to massive consumption of mental and physical resources by employees and therefore result in health problems (e.g., physical pain and sleep disorders) and energy exhaustion (e.g., burnout). Second, job and personal resources are referred to as motivational factors, which could promote work engagement and work performance of employees, and eliminate cynicism. Studies demonstrated that job and personal resources played intrinsic and extrinsic motivational roles in satisfying the basic needs of employees, such as autonomy, relatedness, competence, and professional development, and ultimately led to positive work attitude and high work engagement (Fullemann et al., 2016; Trepanier et al., 2020; Kato et al., 2021). The JD-R model provides a conceptual basis for the NMDI program with regard to the process of improving job and personal resources and includes seven domains of performance, namely, feedback, job control, supervision and guidance, peer support, self-efficacy, self-esteem, and optimism. Hence, we proposed a theoretical framework for an AI-based NMDI program targeting burnout reduction (Figure 1).

According to the theoretical framework for an AI-based NMDI program, the literature review, and the mixed study, the AI-based NMDI program for nurses coping with burnout was developed. The essential components of the NMDI program were discovery (inquire the meaning of the nursing profession, discover an existing job and personal resources of nurses, and help nurses to excavate potential resources that goes unnoticed before), dream (create a nursing dream to change the current status and arouse energy and enthusiasm of nurses), design (construct compelling strategies for turning “dreams” into “reality” and essential elements for resources promotion must be in place), and destiny (practice strategies in normal clinical work with organizational support and share the best practical experience). The detailed contents for the AI-based NMDI programs are listed in Table 2.

**Table 2:** Titles, aims and contents of the AI-based NMDI program sessions.

**Session titles**	**Aims**	**Contents**
1. Inception	- To form an intervention group - To introduce the aims and contents of the program - To construct affirmative themes	- Organize the first meeting and conduct several ice-breaking games to help nurses know each other - Provide intervention manual and introduce the concepts, theory of AI, the process and consideration of the intervention - Form affirmative themes revolving around overall changing agenda *via* “brain storm”
2. Discovery	- To discover personal resources through self-exploration and peer support - To discover organizational resources through managers and peer support	- State of peak experience and explore positive characteristics of nurses - Explore personal strengths *via* character-strengths survey - Communicate with managers and colleagues to list organizational resources that can be used to deal with job-related issues
3. Dream	- To develop self-confidence and a positive self-image - To build positive career dreams	- Gain new insights through reviewing old material, and enhance participants' strengths - Build a career dream using personal strengths and organizational resources and record the dream by mails or painting - Share dreams with group members and construct declarations for the dreams Note: manager participate this process and give suggestion about the dreams
4. Design	- To design the work plan to strengthen job control - To collaborate with colleagues and managers to get organizational resources	- Review the dreams built by participants - Order the goals, personal strengths and organizational resources by importance and figure out work plan to achieve the primary goal with suggestions from managers - Break the work plan into several phases and set short-term goals for each phases. Participants are suggested to record the process of realizing their dreams
5. Destiny	- To form a support team to promote participants' work performance - To organize group meetings to share positive experiences and get supports from managers and colleagues	- Support teams consist of intervenors and managers. Participants can contact with support team to get suggestions. Meanwhile, support teams need to supervise participants to guarantee their performance followed the work plan - Organize online meetings for participants to share their best experiences and discuss with support team and colleagues to cope with difficulties and frustration - Each participant needs to summarize their gains and give out positive concluding statements
6. Keep	- To strengthen personal and organizational resources *via* performance feedback - To create a positive work environment *via* personal competence improvement and good relationships with colleagues and managers - To continue set goals and work plan	- Celebrate the achievements with group members and work out new goals - Explore new personal strengths by sharing successful experiences - Colleagues and managers summarize participants' positive changes and give out positive feedback and expectations - Intervenors review the intervention process and encourage participants and managers to employ AI-based NMDI into their clinical work in future

The proposed AI-based NMDI program is an online and offline mixed burnout intervention program with five weekly sessions and one 4-week session (destiny). Each weekly session lasts for 120–180 min. While, for the 4-week destiny, each week hold a 60–90 min online meeting. Group intervention was employed in all of the sessions. Each group consists of six–eight participants. Managers (e.g., head nurse, vice director, and director of the nursing department) need to participate in each session of the program.

#### Outcome measures

All of the outcome measures were selected based on the theoretical framework of the AI-based NMDI program and were intended to be measured at baseline, after the NMDI program intervention, and 3 and 6 months after the intervention. The outcome measures assess the following variables: performance feedback, job control, supervision and guidance, peer support, self-efficacy, self-esteem, optimism, and burnout. All of the measures employed in the study are established scales with good reliability and validity.

The performance feedback, job control, supervision, and guidance of participants are assessed using the work resources subscales of the job demand and work resources scale (Li et al., 2014). The Colleague Support Scale for the nursing group, developed by Ma and Ye (2007), is employed to evaluate the peer support of participants. The 10-item General Self-Efficacy Scale, developed by Schwarzer et al. (1999), is used to evaluate the self-efficacy status of participants. The 3-item subscale of the Psychological Capital Questionnaire (Luthans et al., 2007) is used to measure the optimism of participants. The 10-item Rosenberg Self-Esteem Scale (Vispoel et al., 2001) is used to measure the self-esteem of participants. The Maslach Burnout Inventory-General Survey (Maslach and Jackson, 1981) is used to measure burnout symptoms experienced by participants.

General information on the sociodemographics and characteristics of both participants and hospitals are collected at baseline. The records written by participants and intervenors are collected after the intervention to support the analysis of intervention effects.

## Discussion

This study was used to develop an AI-based nurse-manager dualistic intervention program to reduce burnout symptoms among nurses. Following the MRC framework for developing and evaluating complex interventions (Moore et al., 2015; Medical Research Council, 2022), a systematic and iterative procedure used for intervention development was described, which involved three steps identifying the evidence base, identifying/developing theory, and modifying process and outcomes. These were done by gathering extensive existing evidence from reviews of the literature, investigations, and interviews with nurses and nurse managers, and applying the job demand-resources model as a theoretical basis to strengthen the design for the intervention. This AI-based nurse-manager dualistic intervention program should be a feasible and effective intervention.

Our study is the first to address nurse burnout by organizing nurses and nurse managers as a whole to discover and utilize the personal strengths of nurses and hospital resources. Compared with current combined interventions (Rodrigues et al., 2018; Kersten et al., 2019), our study focused more on enhancing the value match between nurses and hospitals. There are six sessions for the AI-based nurse-manager dualistic intervention program, and all sessions are very closely interdependent. For instance, the discovery session, which aims to find out personal strengths and organizational resources, acts interdependently with the dream session to build a positive career dream toward work-related burnout symptoms. According to the 4D cycles of AI (Sandars and Murdoch-Eaton, 2017; AI Commons, 2018), the six sessions (inception, discovery, dream, design, destiny, and keep) have direct interrelationships that advance the process of appreciating, envisioning, and creating a “best” future for nurses and reduce their burnout symptoms.

A pilot study and randomized controlled trial (RCT) are recognized as necessary advancements for developing and assessing a complex intervention. A pilot study of the nurse-manager dualistic intervention program needs to be conducted to test the adequacy of the planned methods and procedures, the fidelity of implementation, and the retention of nurse participants, identify potential confounding factors, and evaluate the underlying mechanism for the intervention. The RCT study is highly recommended to measure the effects, safety, cost-effectiveness, and long-term impacts of the dualistic intervention. In addition, a mixed study incorporating both qualitative and quantitative methods could be adopted for evaluating objective outcomes and subjective attitudes of the participants.

The AI-based nurse-manager dualistic intervention program, developed in accordance with the JD-R model, is intended to eliminate the mismatch between nurses and hospitals *via* utilizing the personal strengths of nurses and hospital resources, which, in turn, prevent or alleviate nurse burnout. Thus, performance feedback, job control, supervision and guidance, peer support, self-efficacy, self-esteem, optimism, and burnout of nurses were chosen as the key outcomes. The strength of the relationships among the observed variables and a potential mechanism of the intervention are recommended to be analyzed in RCT studies.

### Limitations

Using theory to design effective personal-organizational dualistic interventions posed a number of challenges, the biggest being the process of blending personal and organizational-oriented interventions. Moreover, another challenge was promoting the feasibility of the intervention to nurse participants and nurse managers. Future studies are suggested to explore the experiences, concerns, and needs of nurses and nurse managers coping with burnout, which could be used to develop these complex interventions. This study focused on the development of this AI-based NMDI program and the recruitment strategies of participants; the effects and generalizability of the program remain uncertain. Further pilot studies and single-center and multicenter studies are recommended to implement and evaluate this complex intervention.

## Conclusion

Following the MRC framework for the development and evaluation of complex interventions, a potentially feasible and effective nurse-manager dualistic intervention program for burnout nurses was developed. Literature reviews, a qualitative and quantitative mixed study, and the job demand-resources model provided the evidence base for intervention development. Future pilot studies and randomized controlled trials need to be employed to evaluate the feasibility, effects, and mechanism of these nurse-manager dualistic intervention programs. Nurses and nurse managers are encouraged to use this dualistic intervention for alleviating burnout after advanced evidence on the effect of the intervention is reported by further studies.

## Data availability statement

The original contributions presented in the study are included in the article/supplementary material, further inquiries can be directed to the corresponding author.

## Ethics statement

Human Research Ethical Approval (No. 2020-R-030) was obtained from the Human Research Ethics Committee of School of Nursing and Rehabilitation, Shandong University. The patients/participants provided their written informed consent to participate in this study.

## Author contributions

Y-FG contributed to the study design, the literature review, data analysis, and writing and revising. X-XW contributed to the literature review, data collection, data analysis, and writing. F-YY and F-YS contributed to data analysis, writing, and revising. MD and Y-NJ contributed to the writing and revising. All authors contributed to the article and approved the submitted version.

## Funding

This study was funded by the National Natural Science Foundation of China (72004120) and the Natural Science Foundation of Shandong Province (ZR2020QG058).

## Conflict of interest

The authors declare that the research was conducted in the absence of any commercial or financial relationships that could be construed as a potential conflict of interest.

## Publisher's note

All claims expressed in this article are solely those of the authors and do not necessarily represent those of their affiliated organizations, or those of the publisher, the editors and the reviewers. Any product that may be evaluated in this article, or claim that may be made by its manufacturer, is not guaranteed or endorsed by the publisher.
